# High-throughput detection of a large set of viruses and viroids of pome and stone fruit trees by multiplex PCR-based amplicon sequencing

**DOI:** 10.3389/fpls.2022.1072768

**Published:** 2022-12-12

**Authors:** Larissa Carvalho Costa, Benjamin Atha, Xiaojun Hu, Kurt Lamour, Yu Yang, Mary O’Connell, Clint McFarland, Joseph A. Foster, Oscar P. Hurtado-Gonzales

**Affiliations:** ^1^ Plant Germplasm Quarantine Program, Animal and Plant Health Inspection Service, United States Department of Agriculture, Beltsville, MD, United States; ^2^ Department of Entomology and Plant Pathology, University of Tennessee, Knoxville, TN, United States; ^3^ Plant Protection and Quarantine - Field Operations, Animal and Plant Health Inspection Service, United States Department of Agriculture, Raleigh, NC, United States

**Keywords:** multiplex PCR, high-throughput sequencing diagnostic, molecular virus and viroid detection, Fruit trees, plant viral infection

## Abstract

A comprehensive diagnostic method of known plant viruses and viroids is necessary to provide an accurate phytosanitary status of fruit trees. However, most widely used detection methods have a small limit on either the number of targeted viruses/viroids or the number of samples to be evaluated at a time, hampering the ability to rapidly scale up the test capacity. Here we report that by combining the power of high multiplexing PCR (499 primer pairs) of small amplicons (120-135bp), targeting 27 viruses and 7 viroids of fruit trees, followed by a single high-throughput sequencing (HTS) run, we accurately diagnosed the viruses and viroids on as many as 123 pome and stone fruit tree samples. We compared the accuracy, sensitivity, and reproducibility of this approach and contrast it with other detection methods including HTS of total RNA (RNA-Seq) and individual RT-qPCR for every fruit tree virus or viroid under the study. We argue that this robust and high-throughput cost-effective diagnostic tool will enhance the viral/viroid knowledge of fruit trees while increasing the capacity for large scale diagnostics. This approach can also be adopted for the detection of multiple viruses and viroids in other crops.

## Introduction

The yield of fruit trees and the quality of their production are heavily dependent on the health status of the trees, which can be affected by many different systemic pathogens. Temperate fruit trees represented by pome (apple, pear, and quinces) and stone fruits (plums, cherries, peaches, nectarines, apricots, and almonds) are commonly affected by many viruses and viroids, which constitute a major threat for fruit production in orchards worldwide (Barba et al., 2015; EFSA Panel on Plant Health et al., 2019; Umer et al., 2019). In addition to yield reduction, the damage caused by viral infections can affect the size, shape, and quality of the fruits, resulting in significant economic losses to all sectors of the production chain (Hadidi and Barba, 2011; Jones and Naidu, 2019; Jones, 2021). Early detection and accurate diagnosis of viral diseases are crucial for developing effective and sustainable management strategies to limit their spread (Ibaba and Gubba, 2020). Conventional methods such as biological indexing, ELISA, and end-point polymerase chain reaction (PCR) based techniques have been historically used for virus detection in fruit trees (Ward et al., 2004; Boonham et al., 2014; Rott et al., 2017; Abd El-Aziz, 2019; Diaz-Lara et al., 2020; Hema and Konakalla, 2021). However, these conventional methods can be hindered by their laborious and time-consuming processing steps, especially when a large set of samples need to be evaluated for the presence of different viruses.

One of the main goals in plant viral diagnosis has been the implementation of effective multiplex methods for the simultaneous detection of multiple targets, which provide speed and cost-efficiency (Pallás et al., 2018; Mehetre et al., 2021). Although the multiplex PCR methods reported thus far have been optimized for the detection of up to nine different plant viruses/viroids in a single assay (Roy et al., 2005; Gambino and Gribaudo, 2006; Gambino, 2015; Kwak et al., 2014; Zhao et al., 2015), the sensitivity for multiple target detection is questionable. Real-time PCR (qPCR) using fluorescence-labelled probes has become very popular for routine plant viral diagnosis due to the improvements in sensitivity and specificity, elimination of post-PCR manipulations, and superior potential for multiplex when compared to regular PCR (Mortimer-Jones et al., 2009; Lin et al., 2013; López-Fabuel et al., 2013; Otti et al., 2016; Minutolo et al., 2020). However, the limited number of available fluorescent reporter dyes has also restricted the number of pathogens that can be detected using multiplex assays. For these reasons, both PCR and real-time multiplex PCR that combine two or more assays is still challenging (Boonham et al., 2014; Pallás et al., 2018; Hema and Konakalla, 2021).

Another diagnostic method that has the potential to detect a large number of plant viruses/viroids in a single assay is DNA microarrays (Boonham et al., 2008; Zhu et al., 2017; Hema and Konakalla, 2021). This technology has been utilized for virus detection in different crops, including fruit trees (Lenz et al., 2008; Engel et al., 2010; Tiberini et al., 2010; Abdullahi et al., 2011; Tiberini and Barba, 2012; Thompson et al., 2014). The major limiting factor of the microarray approach is that the detection process is complex and costly (Zhu et al., 2017; Hema and Konakalla, 2021). Advancements in sequencing technologies have allowed DNA microarrays to be rapidly replaced with high throughput sequencing (HTS) (Boonham et al., 2014; Hema and Konakalla, 2021). The simultaneous detection of multiple viruses, without prior knowledge of their genomic sequences, makes metagenomics-based HTS a very attractive option in the field of plant viral diagnosis (Pecman et al., 2017; Rott et al., 2017; Maree et al., 2018; Villamor et al., 2019; Gaafar et al., 2021; Mehetre et al., 2021). As a result of its potential to provide a complete view of the virome of a given host, HTS has been used to identify and characterize several novel viruses and variants of known viruses of fruit trees with various approaches (Marais et al., 2015b; Villamor et al., 2016; Wu et al., 2017; Diaz-Lara et al., 2018; Liu et al., 2018; Maliogka et al., 2018; Diaz-Lara et al., 2019; Tahzima et al., 2019; Larrea-Sarmiento et al., 2020; Costa et al., 2022).

Since the metagenomics-based HTS approach is gaining more attention for viral diagnostics, there is a need for suitable bioinformatic pipelines and expertise to analyze the large amount of data generated (Pallás et al., 2018; Villamor et al., 2019; Bester et al., 2021). Another important drawback with the detection of plant viruses from metagenomic sequencing datasets is the overabundance of unnecessary plant host reads and the small number of virus-derived reads, especially for plants containing viruses in low titer (Adams and Fox, 2016; Gaafar et al., 2021). Metagenomics-based HTS has become more cost-effective compared with multiple singular conventional tests, however, its cost is still too high to be used as a routine diagnostics tool. To increase the likelihood for detection of viruses even in low titer, targeted sequencing approaches have been proposed (Espindola et al., 2021; Maina et al., 2021).

In this study, a robust, cost-effective multiplex PCR-based amplicon sequencing approach (HiPlex) is described for the simultaneous detection of 34 commonly found viruses and viroids of pome and/or stone fruit trees in over 100 samples. The accuracy, sensitivity, and specificity of this approach were also compared with other detection methods including HTS of total RNA (RNA-Seq) and individual RT-qPCR assays.

## Materials and methods

### Infected fruit tree samples used for HiPlex validation

A total of 24 known virus/viroid-infected fruit trees, which are routinely used as positive controls in virus/viroid PCR-based diagnostics at USDA-APHIS-PPQ-PGQP (Plant Germplasm Quarantine Program), were included for the validation of the customized fruit tree HiPlex. All together, these 24 positive control trees contained 27 viruses and 7 viroids of pomes and stones (**Table 1**). Trees included 13 pome fruit trees (7 apples (*Malus domestica*), 5 pears (*Pyrus communis*), and 1 quince (*Cydonia oblonga*)) and 11 stone fruit trees (6 cherries (*Prunus avium*), 3 peaches *(Prunus persica)*, 1 apricot (*Prunus armeniaca*), and 1 plum (*Prunus domestica*)). One apple tree (*M. domestica*) known to carry no plant viruses or viroids was also included as a negative control in all experiments. All 25 trees ranged in age from 3 to 8 years old, and were grown in ten-gallon pots, in secured screenhouses in quarantine at PGQP, located at Beltsville Agricultural Research Center, East campus, Beltsville, Maryland, USA. Fully developed leaves, collected from at least five different branches per tree, were used for total RNA extractions.

**Table 1:** Viruses and viroids included in this study and the positive fruit tree samples used for each one.

Virus/viroids				Positive samples
Family	Genus	Species	Abrev	
**POME**				
*Avsunviroidae*	*Pelamoviroid*	*Apple hammerhead viroid*	AHVd	PGQP-5,6,7,8
*Betaflexiviridae*	*Capillovirus*	*Apple stem grooving virus*	ASGV	PGQP-5,6,7,8,11
	*Foveavirus*	*Apple green crinkle associated virus*	AGCaV	PGQP-4,5,6,7,12, 14
		*Apple stem pitting virus*	ASPV	PGQP-4, 5,6,7,8,9,12, 13,14
	*Tepovirus*	*Prunus virus T*	PrVT	PGQP-10
	*Trichovirus*	*Apple chlorotic leaf spot virus*	ACLSV	PGQP-6,7,8,9
*Bromoviridae*	*Ilarvirus*	*Apple mosaic virus*	ApMV	PGQP-5
*Luteoviridae*	*Luteovirus*	*Apple luteovirus 1*	ALV-1	PGQP-3
*Partitiviridae*	*Alphapartitivirus*	*Pear alphapartitivirus*	PAPV	PGQP-11
*Phenuiviridae*	*Coguvirus*	*Citrus concave gum-associated virus*	CCGaV	PGQP-4,8
		*Citrus virus A*	CiVA	PGQP-9,13,14
	*Rubodvirus*	*Apple rubbery wood virus 1*	ARW-1	PGQP-13,14
		*Apple rubbery wood virus 2*	ARW-2	PGQP-4,6
*Pospiviroidae*	*Apscaviroid*	*Apple scar skin viroid*	ASSVd	PGQP-7,11
		*Apple dimple fruit viroid*	ADFVd	PGQP-3
		*Apple fruit crinkle viroid*	AFCVd	PGQP-2
		*Pear blister canker viroid*	PBCVd	PGQP-12,14
**STONE**				
*Avsunviroidae*	*Pelamoviroid*	*Peach latent mosaic viroid*	PLMVd	PGQP-23,24
*Betaflexiviridae*	*Capillovirus*	*Cherry virus A*	CVA	PGQP-19,20,21
	*Foveavirus*	*Asian prunus virus 2*	APV2	PGQP-24
		*Asian prunus virus 3*	APV3	PGQP-24
	*Robigovirus*	*Cherry green ring mottle virus*	CGRMV	PGQP-20
		*Cherry necrotic rusty mottle virus*	CNRMV	PGQP-20,21
	*Trichovirus*	*Peach chlorotic leaf spot virus*	PCLSV	PGQP-24
*Bromoviridae*	*Ilarvirus*	*Prune dwarf virus*	PDV	PGQP-17
		*Prunus necrotic ringspot virus*	PNRSV	PGQP-19
*Closteroviridae*	*Ampelovirus*	*Little cherry virus 2*	LChV2	PGQP-20,21
		*Plum bark necrosis stem pitting-associated virus*	PBNSPaV	PGQP-21,24
	*Velarivirus*	*Little cherry virus 1*	LChV1	PGQP-21,25
*Comovirinae*	*Nepovirus*	*Cherry leaf roll virus*	CLRV	PGQP-15,16
*Luteoviridae*	*Luteovirus*	*Nectarine stem pitting associated virus*	NSPaV	PGQP-18
		*Peach associated luteovirus*	PaLV	PGQP-22
*Pospiviroidae*	*Hostuviroid*	*Hop stunt viroid*	HSVd	PGQP-23
*Tymoviridae*	*Marafivirus*	*Nectarine virus M*	NeVM	PGQP-18

### Total RNA extractions

At least 10 mg of fresh tissue from the bottom third section of all collected leaves, including the mid-rib, was used for total RNA extractions of each sample. Total RNA was isolated using either the RNeasy^®^ Plant Mini Kit (QIAGEN) or the GenCatch™ Plant RNA Purification Kit (Epoch Life Science Inc.) following the respective manufacturer’s guidelines. RNA extracts were then quantified using the Qubit™ RNA BR Assay Kit (Invitrogen™) and later stored at -80°C until ready to be processed. A ribonuclease inhibitor (RNasin, Promega, Madison, WI, USA) was added to each RNA extraction (10 units per 50 µL of nucleic acid).

### HTS-based diagnostics through RNA-Seq of fruit tree samples

RNA-seq libraries were prepared from the total RNA extracts of the 25 fruit tree samples using the TruSeq Stranded Total RNA Library Plant Kit (Illumina, Inc.) with Ribo-Zero™ for ribodepletion, according to the manufacturer’s instructions and our current standard operating procedures (Malapi-Wight et al., 2021). After quantification on TapeStation 4200 (Agilent), each library was diluted and equimolarly used to prepare a pool for later sequencing on the Illumina NextSeq 500 (Illumina, Inc.) at the USDA-APHIS PGQP facilities, with a 75-cycle high output sequence kit (1x 86bp, single-end).

Bioinformatic analysis of RNA-seq data was performed using the USDA-APHIS PGQP PhytoPipe workflow (https://github.com/healthyPlant/PhytoPipe). Briefly, after demultiplexing and adapter trimming using bcl2fastq (v.2.20.0.422) Conversion Software 1.8.4. (Illumina, Inc.), the quality of the sequences was assessed using FASTQC (Andrews, 2010). Raw reads were then filtered and trimmed using Trimmomatic (v.0.39) (Bolger et al., 2014) with parameters “Leading:3, Trailing:3, SlidingWindow:4:20, Minlen:36”. Host reads were filtered using a kmer-based method, where the modified KrakenTools (https://githubcom/jenniferlu717/KrakenTools 2021) extracted unclassified and pathogen-derived (bacteria, fungi, viruses) reads based on the Kraken2 algorithm (Wood et al., 2019). These reads were then assembled using *de novo* assembly tool Trinity (v2.8.6) with default settings (Grabherr et al., 2011). The generated contigs were compared to the NCBI viral reference database (Jan 2022) by BLASTn search (Camacho et al., 2009) using a 1e-10 e-value cut-off, and to the Viral Database (RVDB) protein database (v22) (Bigot et al., 2020) by Diamond blastx search (Buchfink et al., 2021) using a 1e-3 e-value cut-off.

### Primer design for the selected viruses and viroids used in the HiPlex

Genome sequences of the targeted viruses and viroids were downloaded from NCBI at the time of the primer design process in July 2021. To account for as much isolate diversity as possible, we also included *de novo* HTS-detected non-public genome assemblies obtained from our own interceptions that originated from various parts of the world (data not published). Consensus sequences were constructed from the alignments (Clustal Omega) using varying levels of degeneracy (50–85%) to account for nucleotide diversity during the primer design process for each of the targeted viruses and viroids. Sequence manipulation and primer designing were done using the Geneious Prime^®^ 2021.1.1 software (Biomatters Ltd). The majority of the primers were designed to span the coat protein gene of the viral genome. Primers targeting the small viroid genomes were designed along the complete genome when possible. In the case of segmented RNA viruses, each molecule was targeted for primer design to increase the likelihood of detection of one or more RNA molecules. Amplicon size was set between 120 to 135 bp in length with partial overlapping covering viral genomic regions of at least 500 bp when possible. All targeted regions were in-silico analyzed by BLASTn search (Camacho et al., 2009) to confirm uniqueness of the amplicon at the species level. A total of 499 primer pairs were designed across the consensus genome sequences of the 34 viruses/viroids of pomes and stone fruit trees. The number of primers designed per virus/viroid was dependent on the targeted genome region size and ranged from 4 to 22 primer pairs. Additionally, a total of 46 primer pairs were designed to target Phaseolus vulgaris alphaendornavirus 1 and 2 genomes (PvEV1 and PvEV2). These viruses are commonly used as internal controls in HTS-based virus detection assays through spike-in of nucleic acids derived from the black turtle soup common bean variety (Kesanakurti et al., 2016). Thus, the total number of primers in the HiPlex was 545. Detailed consensus sequences and primer information can be found in the **Supplementary Table S1**. All primers pairs were randomly organized in four groups and synthesized by Integrated DNA technologies (Coralville, Iowa, USA).

### cDNA synthesis for HiPlex PCR

Total RNA from all fruit tree samples was converted into cDNA using the Maxima H Minus First Strand cDNA Synthesis Kit (Thermo Scientific, Waltham, MA, USA) following the manufacturer’s protocol. Total RNA from the healthy control apple sample was first spiked with 5 µl of total RNA extracted (concentration at 200 ƞg/µL) from common bean cultivar carrying PvEV1 and PvEV2. For cDNA synthesis, 13 µL of total RNA (or up to 5 µg) from each positive fruit tree sample was used along with 1 µL of random hexamers (100 pmol) and 1 µL of 10 mM dNTP mix. Samples were then incubated on a thermocycler at 65°C for 5 minutes and then placed on ice for 3 minutes, a procedure recommended for any template with secondary structures. Each sample then received 4 µL of 5X RT Buffer and 1 µL of Maxima H Minus Enzyme Mix and was incubated on a thermocycler for 10 minutes at 25°C followed by 30 minutes at 50°C and finally 5 minutes at 85°C to terminate the reaction. cDNA generation was determined using the Qubit™ ssDNA Assay Kit (Invitrogen™). The procedure of cDNA synthesis was conducted in four technical replicates for each of the 25 fruit tree samples used during the validation of the HiPlex, thus generating a total of 100 cDNA reactions.

Multiplex PCRs for all cDNA samples were conducted at Floodlight Genomics (FLG) (Knoxville, TN, USA) as previously described (Nguyen-Dumont et al., 2013) using proprietary technology. A total of 2 ul of cDNA template was used per PCR reaction using one of the four pools of primers plus proprietary adaptors. An aliquot of each PCR reaction from all the four pools of primers was then combined (barcoded pool) and gel purified for size selection using the QIAquick gel extraction kit (QiagenTM). The barcoded pool was quantified using the Qubit™ 1x HS dsDNA Assay kit (Invitrogen™).

### Amplicon library preparation, sequencing, and analysis

A single library was prepared from the pooled barcoded amplicons generated from the HiPlex PCR with the 100 cDNA samples (25 unique fruit trees in four replicates) using the KAPA HyperPrep PCR-free kit (Roche Molecular Systems, Inc.) according to the manufacturer’s instructions. The quality and quantity of the library were analyzed using TapeStation 4200 (Agilent) and Qubit 1X dsDNA HS Assay Kit (Invitrogen) respectively. Then the library was quantified again using the KAPA Library Quant Kit (Illumina/ROX Low) (Roche Diagnostics). The final library was loaded at 1.3 pM while spiked with PhiX internal control at 1/3 of the reaction to add diversity to the sequencing run of the NextSeq500. Pooled samples were sequenced bi-directionally with a 300-cycle mid-output sequence kit (2x151, pair-end) in the NextSeq 500 Illumina system at USDA-APHIS-PPQ-PGQP. A similarly prepared library of the exact same pooled amplicons was used to run on the MiSeq Illumina system, loaded at 12 pM, sequenced bi-directionally with a 300-cycle sequence V2 kit (2x151, pair-end).

The bcl files generated by amplicon sequencing were converted to FASTQ using bcl2fastq software (Illumina, Inc.) and assessed for quality using FASTQC (Andrews, 2010). Data was trimmed and demultiplexed, and R1 and R2 reads were merged using PEAR (Zhang et al., 2014). Reads were then filtered by length to minimize the handling of shorter reads that could have been the product of unspecific smaller-than-target amplificon. A minimum read length of 100bp was considered for further analysis. Filtered reads were directly mapped to the virus consensus sequences using Usearch (v11.0.667) (Edgar, 2010) with parameters “-id 0.85 -strand both -usearch_global -blast6out. The mapped reads per virus/viroid per sample were counted with a home-made python script. A threshold based on inflection point (tangent point) from the sorted mapped read numbers from all samples was set for the identification of true and false positive matches for both NextSeq 500 and MiSeq outputs. The specificity and sensitivity of the proposed amplicon sequencing approach for virus/viroid detection in each sample was evaluated based on the number of true positives, true negatives, false positives, and false negatives considering either RNA-Seq or RT-qPCR results as reference $(\mathrm{sp}ecificity=\frac{True\;negative}{True\;negative+false\;positive})$ ; $(sensitivity=\;\frac{True\;positive}{True\;positive\;+false\;negative})$ . An average percentage specificity and an average percentage sensitivity were then calculated considering the average of the individual specificity and sensitivity results for all the samples in a sequencing run.

### RT-qPCR assays of all targeted viruses and viroids included in the HiPlex validation

Total RNA extracts from the 25 fruit tree samples, used in the validation of the HiPlex, were screened by RT-qPCR for the 36 viruses/viroids targeted in this study, including the PvEV1 and PvEV2 internal controls. Among the 36 assays, 15 were published in previous studies (Osman et al., 2014; Katsiani et al., 2018; Diaz-Lara et al., 2020; Diaz-Lara et al., 2022), four assays (Apple stem pitting virus (ASPV), Apple scar skin viroid (ASSVd), Apple stem grooving virus (ASGV), and Pear blister canker viroid (PBCVd)) are from unpublished data (Al Rwahnih, Foundation Plant Services, CA, USA) and the remaining 17 were developed for this study with the online IDT PrimerQuest™ Tool using the same consensus sequences that were generated for the HiPlex primer design (**Supplementary Table S2**). All probes for the new assays were labeled with a FAM reporter dye on the 5’ end and with an Iowa Black Quencher (3IABkFQ) on the 3′ end according to the IDT manufacturer. The efficiency of all newly developed RT-qPCR assays was determined by standard curves from serial dilutions (1:1 to 1:1,000,000) of total RNA extracts of positive controls using three replicates per dilution point. Additionally, the assays were multiplexed with an 18S rRNA assay for RNA quality control using a probe labeled at the 5’ end with ABY reporter dye and at the 3’ end with QSY quencher (USDA-APHIS-PPQ S&T, work instruction, internal report).

All RT-qPCR reactions were run in triplicate on the QuantStudio™ 3 Real-Time PCR System (Applied Biosystems) using either the TaqMan™ Fast Virus 1-Step Master Mix (Applied Biosystems) or the SuperScript™ III Platinum™ One-Step qRT-PCR Kit from Invitrogen (**Supplementary Table S2**). The reaction using TaqMan™ Fast Virus (10 µL final volume) included 2 µL of total RNA and primers and probe (assay mix) concentrations of 900 and 250 nM, respectively. The thermocycler conditions were as follows: 50°C for 5 min, 95°C for 20 s, followed by 40 cycles of 95°C for 3 s and 60°C for 30 s. The reaction using SuperScript™ III Platinum™ (20 µL final volume) included 2 µL of total RNA and primer and probe concentrations of 500 and 100 nM, respectively. The following cycling conditions were used during RT-qPCR with the SuperScript kit: 50°C for 15 min, 94°C for 2 min, followed by 40 cycles of 94°C for 10 s and 60°C for 45 s.

### Application of the HiPlex amplicon sequencing for virus/viroid detection on pome field samples

To test the robustness of the HiPlex developed for the detection of viruses and viroids of pomes and stone fruit tree samples, an additional set of 109 field samples from pome fruit trees was also screened using this new proposed amplicon sequencing approach. These field samples had been previously screened for the presence of the commonly found viruses and viroids of pome species by RT-PCR (Hurtado-Gonzales, APHIS-PPQ-PGQP 2020 internal report). These samples were collected during the summer of 2020 in three different states of the United States: Maryland (three counties (Ann Arundel, Davidsonville, and Frederick), including 3 orchards and 2 retail stores), Virginia (five counties (Montgomery, Nelson, Stephen City, Willis, and Winchester), including 9 orchards and 1 homeowner backyard), and in Geneva, New York. All 13 pome positive controls plus the healthy control sample previously used during the validation of the HiPlex were also included as controls. The final number of assessed samples was then 123 in total. Total RNA extractions of field samples, cDNA synthesis, and multiplex PCR were performed as described above. All RNA extracts from the field samples were spiked with 0.65 µg of total RNA extracted from the common bean carrying PvEV1 and PvEV2. The spike was done to ensure amplification and sequencing of at least the internal control for every field sample. The library for the pooled amplicons was also prepared and quantified as described above, and it was loaded at 2.3 pM containing 10% of PhiX on the NextSeq 500 using a 300-cycle mid-output kit (2x151, pair-end). Bioinformatic analysis was done using the approach mentioned above. Because different RNA extractions were used for the previous RT-PCR and the amplicon sequencing, the discrepancies between the results of these two methods were also investigated by RT-qPCR assays with the same RNA extracts used in the amplicon sequencing.

## Results

### HTS-based detection of viruses and viroids using total RNA

High throughput sequencing of total RNA (RNA-Seq) yielded seven to 29 million high quality reads per sample for the 25 fruit tree samples after quality control trimming (**Table 2**). Raw reads were filtered for host reads removal, and, with few exceptions, the percentage of reads remaining from viruses/viroids were less than 1% of the total number of clean reads (**Table 2**). Detected viruses/viroids mapping information was shown in **Supplementary Table S3**. The minimum percentage of genome coverage was 38.86% but most of them were above 90%. The consensus NCBI Blastn E-value was 0 for viruses and less than 1E-100 for viroids. Eight samples showed a single viral infection while the other samples had mixed infections with up to six viruses/viroids. No viral reads were found in the healthy control using our current bioinformatic pipeline (PhytoPipe).

**Table 2:** Comparison of the results for virus and viroid detection using RNA-Seq of total RNA, amplicon sequencing and individual RT-qPCR assays.

Sample	Crop	Total number of clean reads		Virus/viroids	Reads Mapped to the target viruses				Ct values RT-qPCR assays
					Total	%	Total	%	
		RNA-Seq	Amplicon Sequencing^1^		RNA-Seq		Amplicon Sequencing^1^		
**POME**									
PGQP-1	Apple	12,866,464	233,943	PvEV1	NA	–	28,767	17.78	20.23
				PvEV2	NA		12,831		21.07
PGQP-2	Apple	21,135,806	287,167	AFCVd	709	0.003	50,054	18.32	18.53
PGQP-3	Apple	27,855,973	298,280	ADFVd	7,467	0.016	70,805	27.73	16.96
				ALV-1	4,494		11,909		22.22
PGQP-4	Apple	21,790,801	410,250	AGCaV	6,118	0.147	2,210	51.95	23.70
				ARW-2 (Sg L, M, and S)	367		24,266		24.05
				ASPV	21,243		30,794		24.62
				CCGaV	4,287		155,842		25.86
PGQP-5	Apple	16,184,442	312,967	ACLSV	5,727	2.611	29,603	69.41	17.81
				AGCaV	3,141		21,082		24.99
				ApMV (RNA 1,2, and 3)	390,712		86,276		19.92
				ASGV	3,352		40,220		17.67
				ASPV	4,812		40,049		18.86
PGQP-6	Apple	12,693,083	423,595	ACLSV	604	0.343	29,563	57.66	22.31
				AGCaV	19,060		154,022		20.02
				ARW-2 (Sg L, M, and S)	3,770		21,974		17.93
				ASGV	912		23,885		22.95
				ASPV	19,231		14,783		24.01
PGQP-7	Apple	26,750,019	302,815	ACLSV	362	3.299	19,717	26.73	26.63
				AGCaV	13,457		5,542		23.28
				AHVd	371,517		8,365		22.69
				ASGV	2,351		9,297		20.29
				ASSVd	480,298		25,965		23.51
				ASPV	14,418		12,088		19.09
PGQP-8	Apple	12,454,270	432,835	ACLSV	286	0.183	28,140	39.38	24.52
				AGCaV	5195		3,511		20.01
				AHVd	728		2,480		22.81
				ASGV	581		1,752		23.75
				ASPV	5,710		17,214		27.35
				CCGaV (RNA1 and 2)	10,330		117,374		27.02
PGQP-9	Quince	20,431,230	234,300	CiVA (RNA1 and 2)	33,645	0.165	39,070	16.67	20.55
PGQP-10	Pear	9,439,556	255,436	PrVT	4,451	0.047	79,663	31.19	19.37
PGQP-11	Pear	23,396,885	373,392	ASGV	2,462	0.160	5,073	72.74	22.15
				ASSVd	829		21,009		25.17
				PAPV (RNA1 and 2)	36,530		245,505		24.15
PGQP-12	Pear	25,212,858	221,922	AGCaV	11268	0.139	6,189	24.56	26.61
				ASPV	19613		20,255		26.13
				PBCVd	4110		28,052		20.45
PGQP-13	Pear	16,696,786	473,912	AGCaV	–	0.578	33,823	68.289	Undetermined
				ARW-1 (Seg L, M, and S)	30,747		56,507		28.14
				ASPV	24,685		11,833		27.72
				CiVA (RNA1 and 2)	41,000		221,468		17.75
PGQP-14	Pear	23,447,233	398,001	AGCaV	450	0.158	2,482	58.89	25.62
				ARW-1 (Sg L, M, and S)	1,066		21,791		26.60
				ASPV	348		819		28.25
				CiVA (RNA1 and 2)	30,471		185,977		17.39
				PBCVd	4,824		23,318		20.56
**STONE**									
PGQP-15	Cherry	22,528,836	193,377	CLRV (RNA1 and 2)	3,124,397	13.868	94,131	48.68	16.68
PGQP-16	Cherry	29,150,383	232,111	CLRV (RNA1 and 2)	4,171,687	14.311	108,438	46.72	16.68
PGQP-17	Cherry	7,282,845	348,095	PDV (RNA1, 2, and 3)	403,008	5.534	265,094	76.16	18.15
PGQP-18	Cherry	29,380,924	244,514	NeVM	89	0.016	35,293	61.33	22.68
				NSPaV	4,757		114,675		24.14
PGQP-19	Cherry	18,776,746	400,841	CVA	9,797	0.092	114,113	75.50	21.42
				PNRSV (RNA 1 and 2)	7,428		188,507		25.01
PGQP-20	Cherry	25,920,825	427,468	CVA	35,423	0.821	127,034	80.67	19.50
				CGRMV	115,552		121,821		21.06
				CNRMV	7,596		13,638		27.48
				LChV2	54,191		82,390		24.46
PGQP-21	Cherry	17,698,398	288,447	CVA	10,865	0.399	36,885	70.24	23.59
				CNRMV	6,226		99,960		27.17
				LCV1	14,293		779		32.04
				LCV2	27,343		22,442		28.73
				PBNSPaV	11,954		42,545		31.38
PGQP-22	Peach	15,187,524	89,550	PaLV	952	0.006	60,035	67.04	17.73
PGQP-23	Peach	10,105,614	165,184	HSVd	281	0.178	13,392	19.10	23.32
				PLMVd	17,747		18,158		14.29
PGQP-24	Peach	12,781,779	450,466	APV2	30,919	0.842	5,005	74.79	20.87
				APV3	28,712		143,638		17.45
				PBNSPaV	21,409		65,736		28.99
				PCLSV	6674		98,038		19.20
				PLMVd	19,941		24,490		12.62
PGQP-25	Plum	23,996,154	184,744	LChV1	31,195	0.130	–	–	23.48

^1^Amplicon Sequencing results of the run conducted in NextSeq Illumina platform.

### Virus and viroid detection through HiPlex amplicon sequencing on two Illumina sequencers

**Figure 1:**
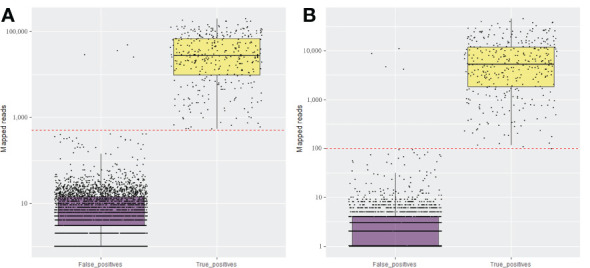
Total number of mapped reads for the expected (True positive) and non-expected (False positive) viruses and viroids in the set 25 fruit tree samples using amplicon sequencing conducted in NextSeq **(A)** and MiSeq **(B)** Illumina systems. The red dotted line is the established threshold to consider the sample as positive.

From the total of 56.45 million paired-end reads generated by the pooled amplicon sequencing of the 100 fruit tree samples (25 unique fruit trees in 4 replicates) using the NextSeq 500 platform, ~52 million paired-end reads were kept after merging and filtering. Amplicon sequencing performed using the MiSeq yielded a total of 9.5 million paired-end reads and 6.5 million paired-end reads were retained after quality control. The reads were also filtered by size for a minimum of 100 bp in length, which corresponded to 80 and 85% of the total high-quality raw reads in the NextSeq and the MiSeq runs, respectively. The same libraries were run on the MiSeq in conjunction with the NextSeq to confirm the robustness of HiPlex amplicon sequencing on a smaller-scale sequencer. Analysis of the four replicates of each sample provided consistent results in both sequencing platforms (**Supplementary Table S4**). Although the MiSeq generated about seven times less sequence data compared to the NextSeq, both runs provided comparable results for the detection of viruses/viroids, with a similar percentage of reads mapped to the target viruses/viroids for both platforms (**Supplementary Table S4**). The majority of the true positive samples had over 5,000 and 1,000 reads mapped to the genome of the expected viruses/viroids in NextSeq and MiSeq outputs, respectively (**Table 2** and **Figure 1**). A threshold of 500 reads in NextSeq and 100 reads in MiSeq were established for considering a sample as positive. Based on these thresholds, the amplicon sequencing approach showed high specificity (99.87%) compared to RNA-Seq. The only false positive (non-expected virus) identified above the threshold was in the case of AGCaV in the four replicates of the pear sample PGQP-13 (**Supplementary Table S4** and **Figure 1**).

A high proportion of the generated reads derived from the amplicon sequencing approach were from the targeted virus/viroid genomes, reaching up to 81% of the total number of reads in a mixed infected cherry sample (PGQP-20) (**Table 2**). The sensitivity of the high throughput amplicon sequencing to detect the viruses and viroids present in the fruit tree samples was 97.83% when compared to the RNA-Seq detection approach. With exception of LChV1, detected in the sample PGQP-25 through RNA-Seq, the amplicon sequencing runs in the NextSeq and the MiSeq were successful at detecting all the target fruit tree viruses/viroids under study (**Table 2** and **Supplementary Table S4**). For the multipartite viruses included in this study, the amplicon sequencing was able to detect all or most of their segments. To avoid false positives, our recommendation is to consider as positive those samples where more than one segment of the virus is detected.

**Figure 2:**
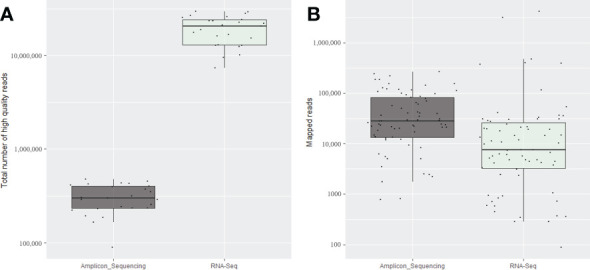
Average of the total number of high-quality reads **(A)** and total number of mapped reads for the expected viruses and viroids **(B)** obtained for the four replicates of each 25 fruit tree samples using the amplicon sequencing conducted on the NextSeq and for the same samples using total RNA-Seq.

The total number of clean reads obtained per sample in total RNA-Seq run was much higher compared with amplicon sequencing, ranged from 20 (PGQP-17) to 125 (PGQP-18) times higher (**Table 2** and **Figure 2A**). However, as the majority of the RNA-Seq reads were from the host genome, the absolute number of reads mapped to the targeted viruses/viroids was, in general, higher using the HiPlex amplicon sequencing approach compared with RNA-Seq (**Table 2** and **Figure 2B**).

### Virus and viroid detection through individual RT-qPCRs

All viruses and viroids detected in the 25 fruit tree samples by RNA-Seq or HiPlex amplicon sequencing were also successfully detected in the individual real time RT-qPCRs assays with the exception of AGCaV in sample PGQP-13. The averages Cq values obtained for each virus or viroid present in each of the 25 fruit tree samples are shown in **Table 2**. The efficiency of the reaction for each of the 17 new developed assays (ADFVd, AFCVd, AGCaV, AHVd, ALV-1, APV2, APV3, CLRV, CiVA, HSVd, PaLV, PCLSV, PLMVd, PAPV, PrVT, PvEV1, and PvEV2) was between 90 to 107% with a ΔRN threshold of 0.1, which is within the range of 90-110% acceptable values for RT-qPCR efficiency (**Supplementary Figure S1**).

### Validation of HiPlex amplicon sequencing using pome field samples

**Figure 3:**
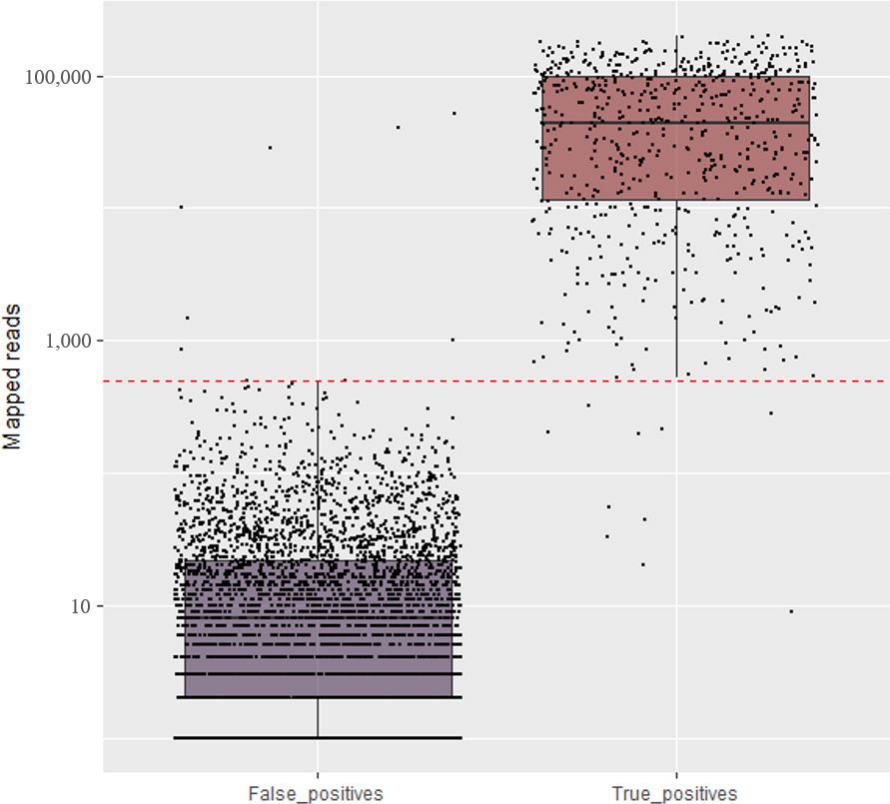
Total number of mapped reads for the expected (True positive) and non-expected (False positive) viruses and viroids in the set of field samples using amplicon sequencing conducted in NextSeq. The red line is the established threshold to consider the sample as positive.

A total of 82 paired-end million reads of sequence data was generated with the amplicon sequencing, for which 67% of the 56,863,033 million high-quality reads (≥100 bp in length) were from the expected viral reads. Similar to the validation of the HiPlex using known positive control samples, high number of reads from each field sample were mapped to the expected virus/viroid genome consensus sequences (**Supplementary Table S5**, **Figure 3**). The two spike-in controls alphaendornaviruses were identified in all field samples with 50% or more of the viral reads mapped to these controls (**Supplementary Table S5**). Nine of the 109 field samples were identified by the HiPlex as single viral infection. In addition, Field-15, 12, 8, 17, 9, 3, and 1 sample(s) were detected as mixed infected by two, three, four, five, six, seven, and eight viruses/viroids respectively (**Supplementary Table S5**). Overall, 74 samples were detected to carry one or more virus/viroid. No viruses/viroids were identified in the other 35 field samples with the exception of PvEV1 and PvEV2 spike-in controls. The virus and viroid composition from all 13 positives plus the healthy control included in the sequencing run of field samples was similar to that of the previous mentioned validation run with the NextSeq (**Supplementary Table S5**).

The amplicon sequencing was effective at detecting, in a single sequencing run, almost all viruses and viroids previously identified by individual RT-PCRs in the set of pome field samples (**Supplementary Table S5**). Considering the threshold of 500 reads, and the RT-PCR or RT-qPCR results as reference, a total of 356 truly positive matches were identified across all virus/viroid identified per sample. Seven false positives were identified for ASGV (1,485 reads), ACLSV (10,170 reads), ASPV (856 reads), ASPV (52,089 reads), AGCaV (28,722 reads), AGCaV (1,013), and AGCaV (40,533) in the samples Field-29, 104, 42, 108, 29, 95, and 104 respectively (**Supplementary Table S5**). Ten false negatives were identified for ARWV-2 (44 reads), ARWV-2 (325 reads), ASGV (280 reads), ASGV (20 reads), ACLSV (55 reads), AGCaV (33 reads), AGCaV (9 reads), AGCAV (212 reads), AGCaV (202 reads), and AGCaV (198 reads) in the samples Field-84, 91, 61, 88, 45, 42, 57, 61, 84, and 107 respectively (**Supplementary Table S5**). Therefore, the specificity and sensitivity of this amplicon sequencing for virus/viroid detection compared to RT-PCR/RT-qPCR was 99.60% and 98.97%, respectively.

## Discussion

Reliable and rapid plant viral diagnostic procedures are of great importance in plant quarantine and certificate programs to ensure the safe exchange of germplasm across countries and for the production of healthy plant material with high yields for growers (Pallás et al., 2018). In recent years, due the exponential increase in information arising from modern sequencing technologies, high-throughput screening for multiple viruses and viroids is gaining more attention (Hema and Konakalla, 2021). In this study, a multiplex PCR coupled with HTS was evaluated and proven to be effective for the simultaneous detection of 34 pome and stone fruit tree viruses/viroids in more than 100 known positive control and field-originated samples. We included only RNA viruses in this HiPlex because they comprised the most important plant pathogens for these fruit trees, however our methodology could easily be adapted for detecting DNA viruses with nucleic acids going straight to the HiPlex PCR.

The comparison of the results of the proposed multiplex PCR-based amplicon sequencing and the RNA-Seq methods showed substantial agreement for the detection of viruses and viroids in the 25 fruit tree samples analyzed (**Table 2**). By allowing a deep characterization of the virome of a given sample, the RNA-Seq method has been increasingly used in plant virology in the past decade and has shown to be powerful and critical in the detection of new viruses (Marais et al., 2015a; Villamor et al., 2016; Wu et al., 2017; Diaz-Lara et al., 2018; Liu et al., 2018; Diaz-Lara et al., 2019; Tahzima et al., 2019; Larrea-Sarmiento et al., 2020; Costa et al., 2022). However, RNA-Seq reads of an infected sample are predominantly composed of host sequences with only a minor fraction of viral sequences. The sequencing of unused large amounts of host-derived reads in RNA-Seq reduces the number of samples to be handled and sequenced per run, making the process costly for its use as a routine diagnostic tool in quarantine and certification programs. The total number of host reads generated per sample through RNA-Seq ranged from 85.69% to 99.9% (PGQP-16 and PGQP-2 respectively, **Table 2**). Conversely, the amplicon-based approach presented here generated as much as 80% of the reads from the targeted viruses and viroids (PGQP-20, **Table 2**).

An advantage of the proposed amplicon sequencing approach is therefore the capacity for increasing the number of samples to be simultaneously screened for multiple viruses/viroids in a single sequencing run. This approach uses a highly streamlined and less expensive and time-consuming workflow of multiplex PCR, library preparation and sequencing, followed by simplified bioinformatics analysis that focus only on the targeted virus/viroid genome sequences included in the designs of the multiplex. The reduction of cost, labor, and time makes the method an attractive option for multiplex detection of pomes and stone fruit tree viruses and viroids. Furthermore, the comparable results for virus/viroid detection obtained for the proposed amplicon sequencing using either the NextSeq 500 or MiSeq (**Supplementary Table S4**) strongly indicates that this approach is very flexible since similar results were obtained with as little as 6.5 million paired-end reads of data from the MiSeq run. To date, the Illumina MiSeq is one of the most popular platforms among different facilities due to its relatively low cost and low error rate. Thus, depending on the number of samples to be analyzed, the MiSeq run is a cost-effective option. The validation run performed on the MiSeq during this study was about half of the cost of the NextSeq run. The HTS library cost is also reduced to one single pooled indexed amplicon using the Hyperprep kit rather than individual TruSeq indexed libraries for each sample that need to be normalized before being pooled and sequenced. The cost per sample tested in the current HiPlex is variable depending on the used sequencing platform reaching between 2-4X less expensive than individual detection using RT-qPCRs. If compared to the regular HTS metagenomic approach for virus/viroid detection, the cost per sample on the HiPlex is about 14X less costly.

Targeted HTS approaches have been commonly applied to human or animal viruses (Nguyen-Dumont et al., 2013; Briese et al., 2015; Wylie et al., 2015; O’Flaherty et al., 2018; Tan et al., 2018; Grubaugh et al., 2019). Fewer examples are found in the literature applied to plant viruses. In recent years the use of this approach has also been increased in plant virology for different purposes, whether for exploring the genetic diversity of virus populations (Kinoti et al., 2017b; Kinoti et al., 2017a; Kinoti et al., 2018; Shaffer et al., 2021; Shaffer et al., 2022) or for improving the limit of detection of HTS-based virus identification (Espindola et al., 2021). Moreover, targeted HTS by sequencing viral amplicons has also been recently reported for simultaneous detection of four faba bean viral species from a multiplex PCR within a 1e-8 serial dilution (Maina et al., 2021). However, no targeted sequencing approaches had covered a wide range of plant viruses in a single multiplex panel to date.

This research investigated the sequencing of a single pool of indexed amplicons from the ultra-high multiplexed PCR of 25 fruit tree samples, which included 545 primers pairs for 36 different viruses and controls, showed comparable sensitivity and specificity for virus/viroid detection with HTS of total RNA (RNA-Seq) and individual RT-qPCR even when RT-qPCR results indicated lower viral titer (low Ct value) due to the late amplification results (**Table 2**). In addition, some viruses/viroids were detected at a more reliable significance level (expressed as higher number of mapped reads) during the multiplex amplicon sequencing when compared to HTS of total RNA-seq (**Table 2**).

Viruses/viroids with RNA genomes, have a great potential for high genetic variability due to their high mutation rates, up to a million times higher than their hosts (Duffy, 2018; Rubio et al., 2020). Therefore, diagnostic procedures must be optimized to cover the maximum number of variants of a particular virus/viroid while always differentiating them from other viruses. To achieve broad viral strain coverage in the amplicon sequencing approach evaluated in the present study, degenerated primer pairs were designed in more conserved genomic regions considering different variants of each targeted virus and viroid (**Supplementary Table S1**). With exception of LChV1 in sample PGQP-1 (**Table 2**), all the other expected viruses and viroids in the 25 fruit tree samples were successfully amplified by multiplex PCR and generated a high number of reads in amplicon sequencing (average of ~35,000 in the NextSeq 500). One possible explanation regarding why LChV1 was missed in sample PGQP-1 during the amplicon sequencing approach is the high genetic variability that has been reported among the isolates of this viral species in stone fruit trees (Tahzima et al., 2019). New primers should be designed to capture more variants of this virus in future multiplex PCRs. This is another advantage of this multiplex PCR-based targeted HTS approach, in which primers can be easily removed or added without disruption, generated improved iterations of the multiplex PCR. Additionally, with high enough degeneracy of primers in conserved regions, it is possible to even amplify potential new viruses, which is a limitation of targeted sequencing compared with RNA-Seq (Kurt Lamour personal communication).

The major challenge of any multiplex PCR-based strategy is not only the amplification bias but possible cross-amplification due to the numerous different primers in a single reaction. There is also a risk of cross-contamination among the samples within a sequencing run. The analytical sensitivity will depend on the contamination threshold fixed for the run (Massart et al., 2022). Indeed, unexpected viral background amplifications were observed but in irrelevant number of reads that, in general, fell below our threshold of 500 and 100 reads to consider a sample as a true positive for a specific virus or viroid on NextSeq and MiSeq runs, respectively (**Figure 1** and **Figure 3**). False positives were identified specially in some cases of AGCaV. This virus has been described as a potential putative distinct foveavirus, but because of the high nucleotide similarity and genome organization with ASPV, it is also considered a variant of ASPV (James et al., 2013). Indeed, all non-expected AGCaV in the first and second amplicon sequencing panels were identified in samples that also carry ASPV [PGQP-13 (**Supplementary Tables S4, S5**)], Field-29, 95, and 104 (**Supplementary Table S5**). It is possible that some primers targeting AGCaV in the multiplex reaction probably also amplified some strains of ASPV. This could also be a reason why AGCaV was detected only by RT-qPCR (Field-57, 61, 84, and 107 (**Supplementary Table S5**)). The degenerated primers designed in this study for AGCaV RT-qPCR assays could also be amplifying ASPV strains present in those samples.

High agreement for virus detection was also observed between the amplicon sequencing and the individual RT-PCR/RT-qPCRs results for the 15 different viruses/viroids found in a set of pome samples collected in different orchards (**Supplementary Table S5**). This panel included a total of 123 samples (109 field samples and 14 positive controls used in the validation step of the amplicon sequencing run), which demonstrated the potential for large-scale detection of multiple viruses in multiple samples in a single sequencing run with a more affordable price than the regular RNA-Seq. Currently our in-house procedures for virus/viroid diagnostics using an RNA-Seq approach allows for a maximum of 24 samples to be sequenced per run using a 75x1 single-end approach (approximately 40 Gb or 500 million reads total). The consistency of the results during the validation of this multiplex using the positive samples supports the accuracy and reproducibility of the method for virus/viroid detection. In addition to AGCaV, a few more discrepancies were observed between the amplicon sequencing and individual RT-PCR/RT-qPCRs results for the 15 different viruses/viroids in a set of pome samples collected in different orchards (**Supplementary Table S5**). In the same way as for the other diagnostic methods, the use of replicates could minimize the occurrence of false positives and false negatives. Moreover, different mapping parameters could cause more stringent mapping or loose mapping.

In this study, 17 new RT-qPCR assays were developed when available tests were non-specific or absent. These assays can be useful in quarantine programs and fruit tree industry for the detection of virus-infected material. The individual RT-qPCRs used were efficient to detect all viruses and viroids under study, however, designing RT-qPCR primers for virus/viroid detection is not a trivial task due to the great genetic variability among strains of a same species. For some viruses, such as in the case of APV2, just one of the five newly designed primer pairs was able to amplify the virus in sample PGQP-24. This means that if just one primer pair and probe are used for routine virus diagnosis, some divergent strains that may be present in some samples could be missed in RT-qPCR assays. Multiplex RT-qPCR with different primers and probes targeting the same virus have been proposed to capture the genetic variability within viral species (Diaz-Lara et al., 202, Al Rwahnih, unpublished). In the proposed amplicon sequencing, multiple primer pairs were designed for each virus. For tripartite genome viruses, such as ApMV, ARWV-1, PDV, and PNRSV, the number of primers included in the multiplex PCR was around 30. For ARWV-2, which is composed of five RNA segments, 60 different primer pairs were designed and included in the multiplex. The large number of primer pairs used reduces the possibility of false positives.

Overall, the multiplex PCR-based amplicon sequencing approach evaluated here is unlike any other amplicon-based sequencing project applied for the detection of a wide range of viruses/viroids. The method allowed for the accurate detection of high and low titer viruses, reduced the background noise from host plants, and improved the reliability of HTS-based virus detection. Our approach offers the opportunity for the rapid diagnosis of multiple viruses in a large set of samples in a single sequencing panel, which ultimately can reduce processing time, labor, and cost associated with virus detection in quarantine centers or in certification programs, in which numerous accessions are annually tested for multiple regulated viruses and viroids. This approach can be adopted for the detection of numerous viruses and viroids in other crops, including DNA viruses. Furthermore, the amount of sequencing data generated on the specific regions of the virus/viroid genomes can facilitate population genetic studies at a large scale.

## Data availability statement

The data presented in the study are deposited in NCBI repository (https://www.ncbi.nlm.nih.gov/), accession number PRJNA896155.

## Author contributions

OH-G and KL conceived the original idea of the project. LC, BA, XH, KL, and OH-G planned the experiments. LC, BA, KL, YY, and MO carried out the experiments. LC, BA, XH, and OH-G analyzed the data and contributed to the interpretation of the results. LC wrote the manuscript. All authors provided critical feedback to shape the research and agreed to the final version of the manuscript. All authors contributed to the article and approved the submitted version.
