# Supplementary material for: High-throughput detection of a large set of viruses and viroids of pome and stone fruit trees by multiplex PCR-based amplicon sequencing
Source: Front Plant Sci. 2022 Dec 12;13:1072768. doi: 10.3389/fpls.2022.1072768 (PMC9791224; doi:10.3389/fpls.2022.1072768)

Figure S1 Standard Curves from serial dilutions of total RNA extract from postive controls for the 17 new developed RT-qPCR assays

(A) Apple dimple fruit viroid

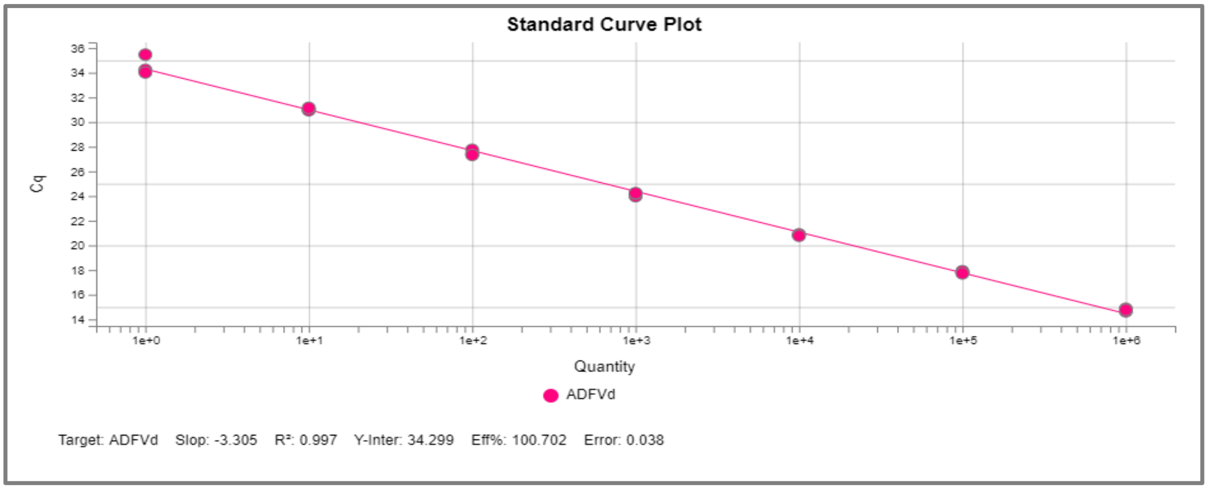

(B) Apple fruit crinkle viroid

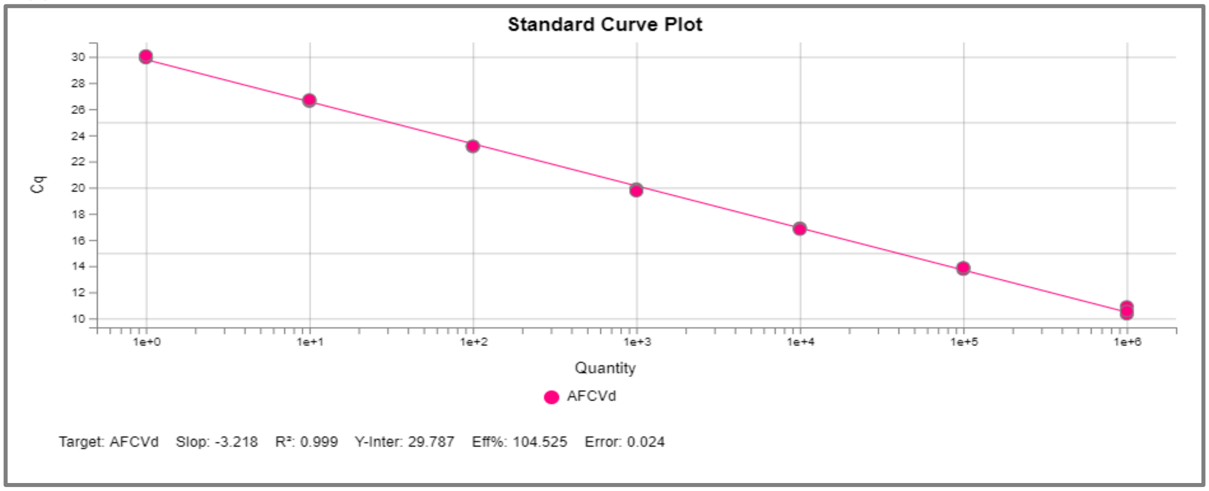

(C) Apple green crinkle associated virus

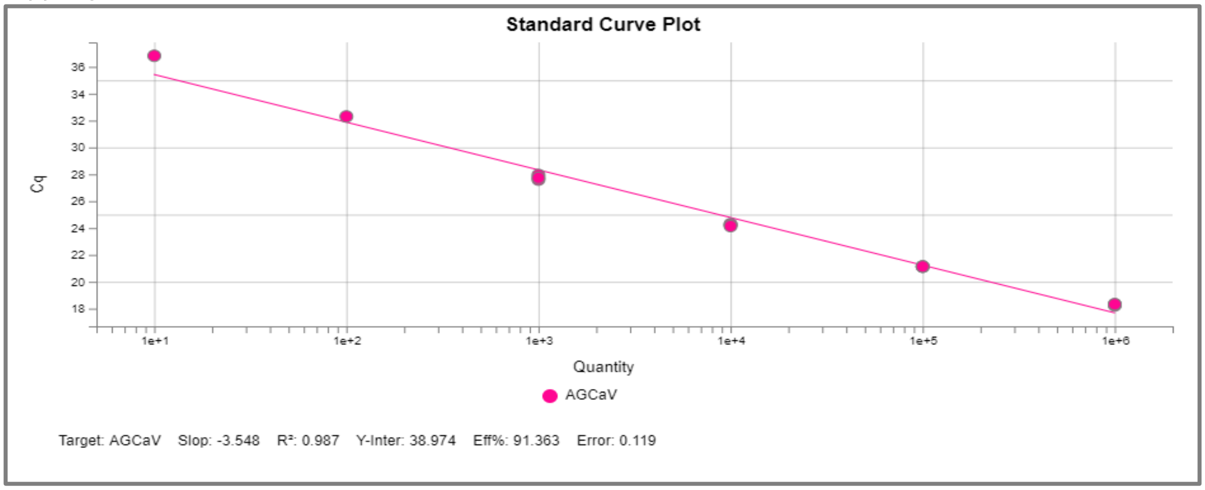

(D) Apple hammerhead viroid

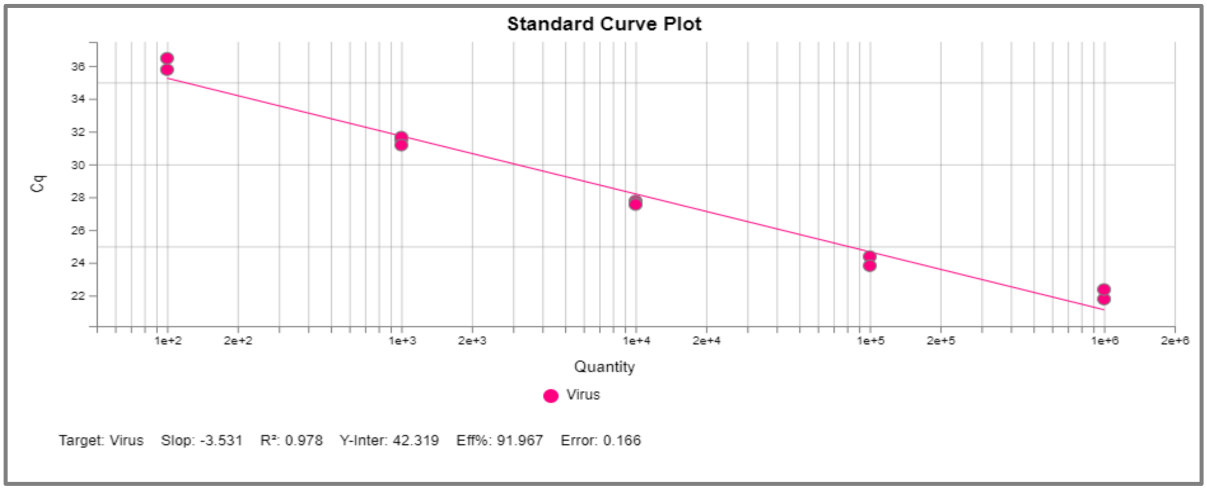

(E) Apple luteovirus 1

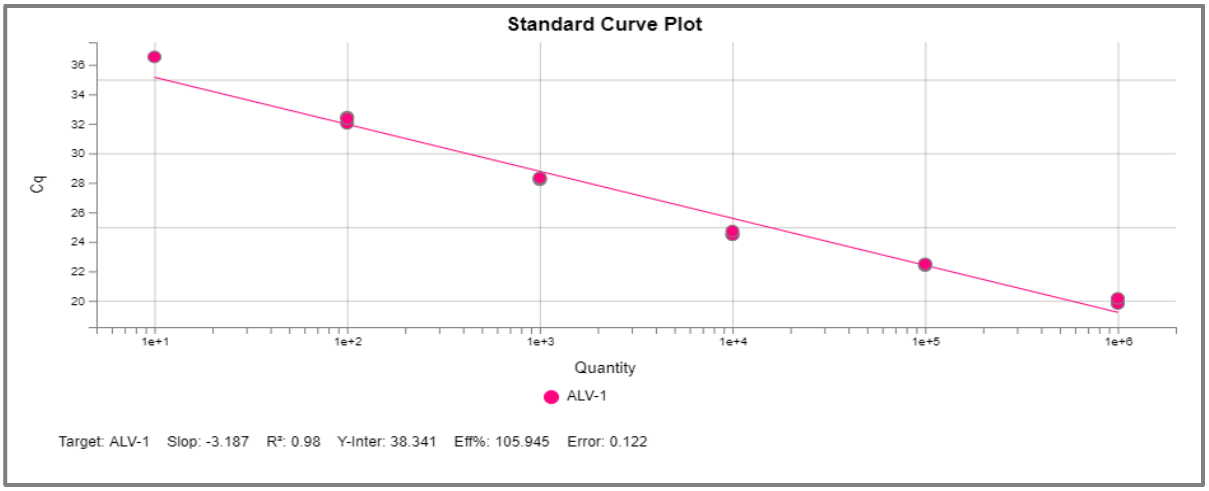

(F) Asian prunus virus 2

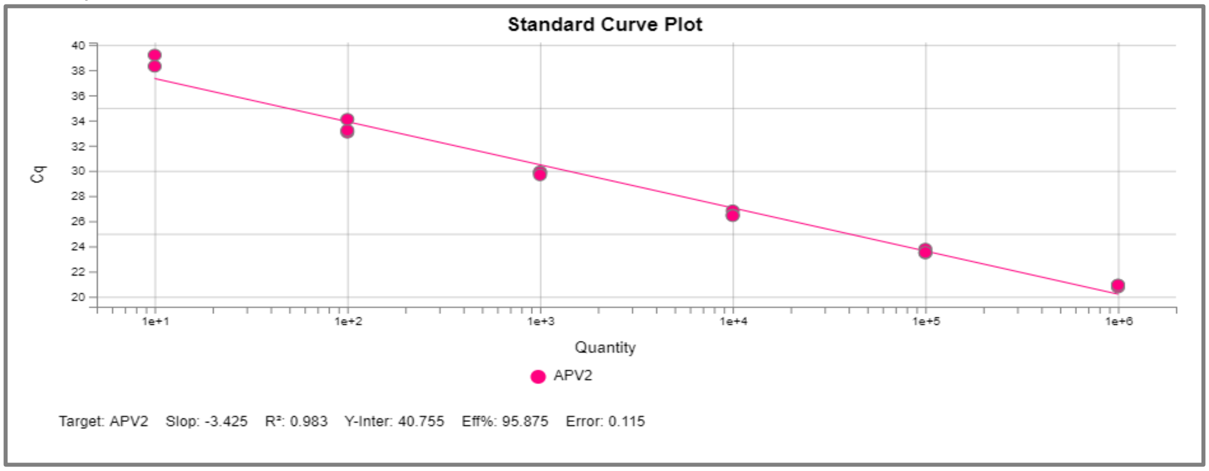

(G) Asian prunus virus 3

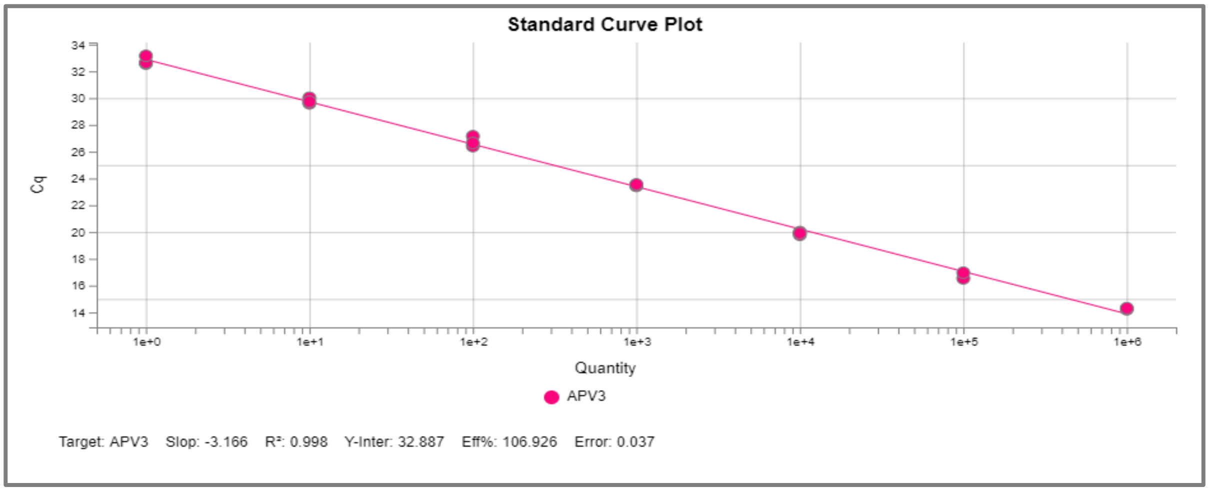

(H) Cherry leaf roll virus

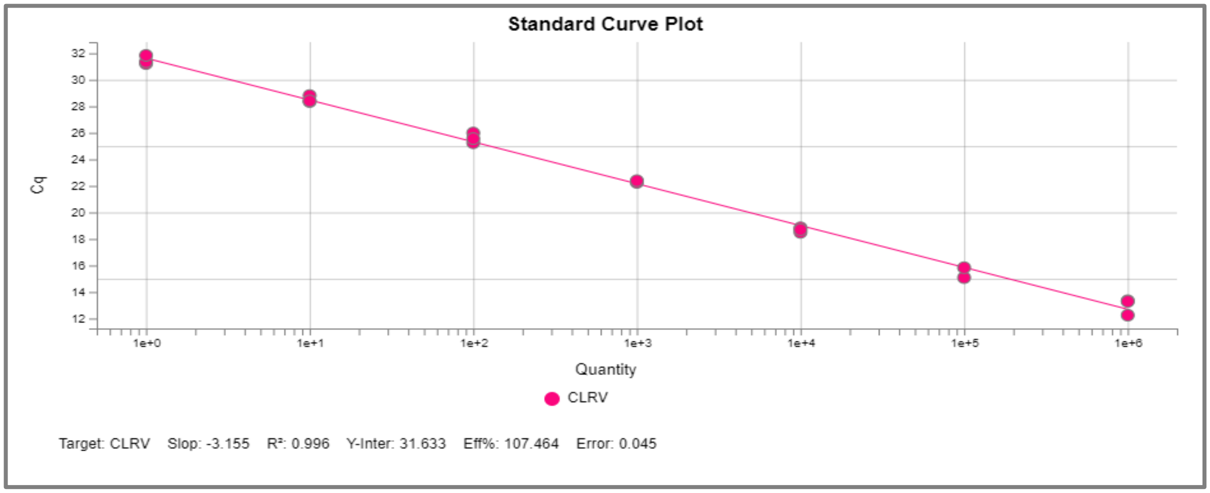

(I) Citrus virus A

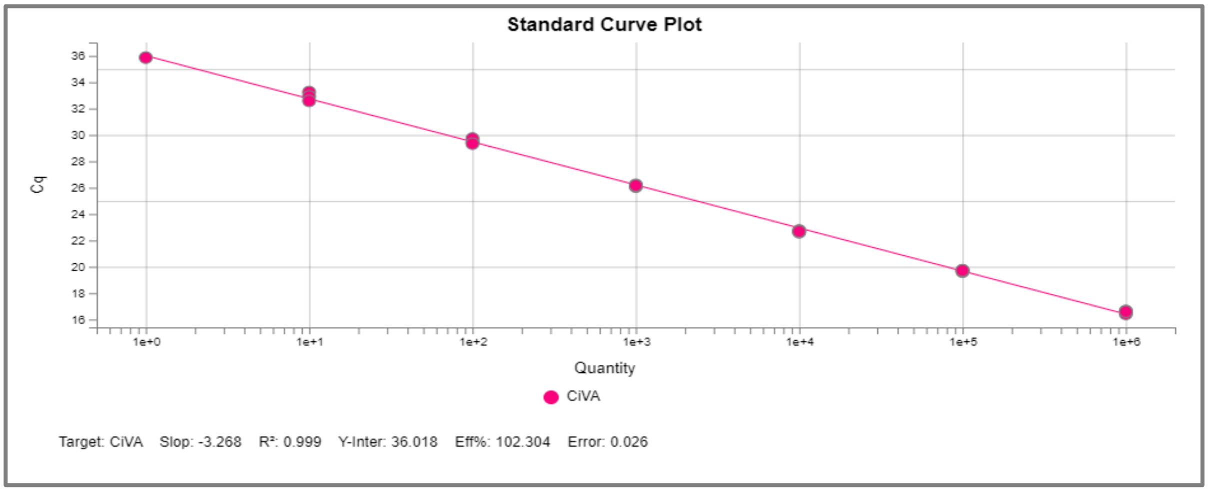

(J) Hop stunt viroid

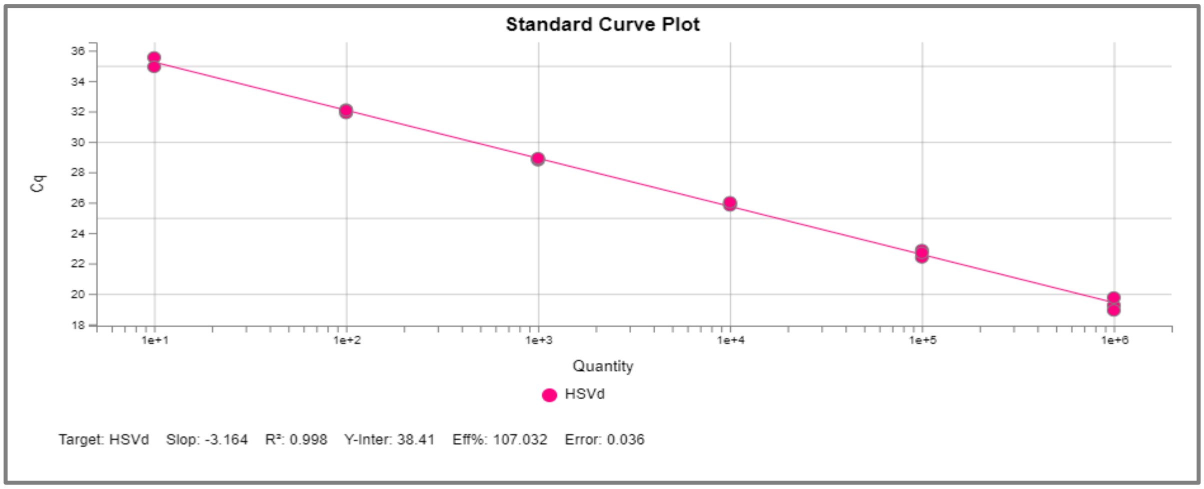

(K) Peach associated luteovirus

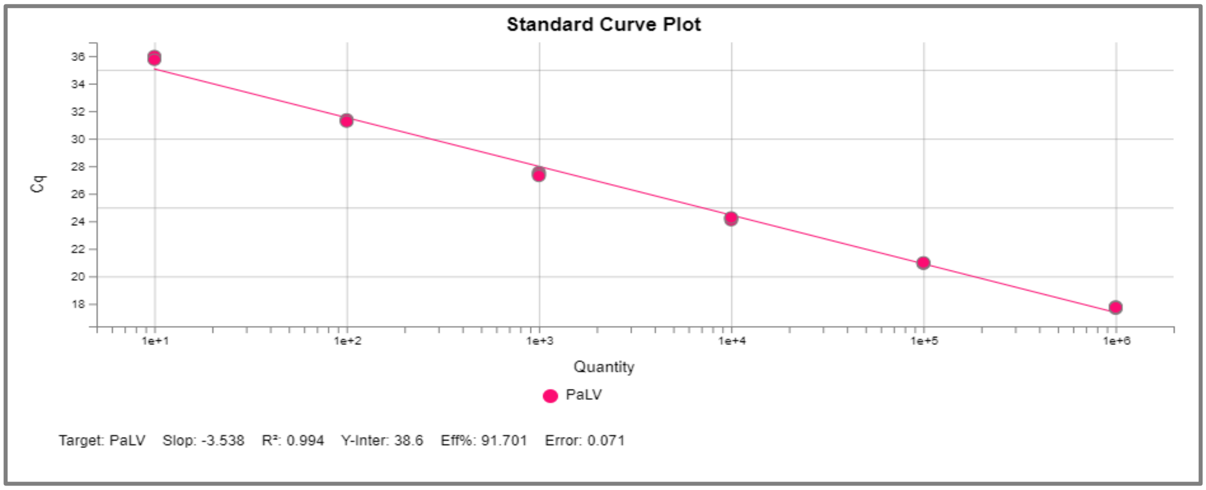

(L) Peach chlorotic leaf spot virus

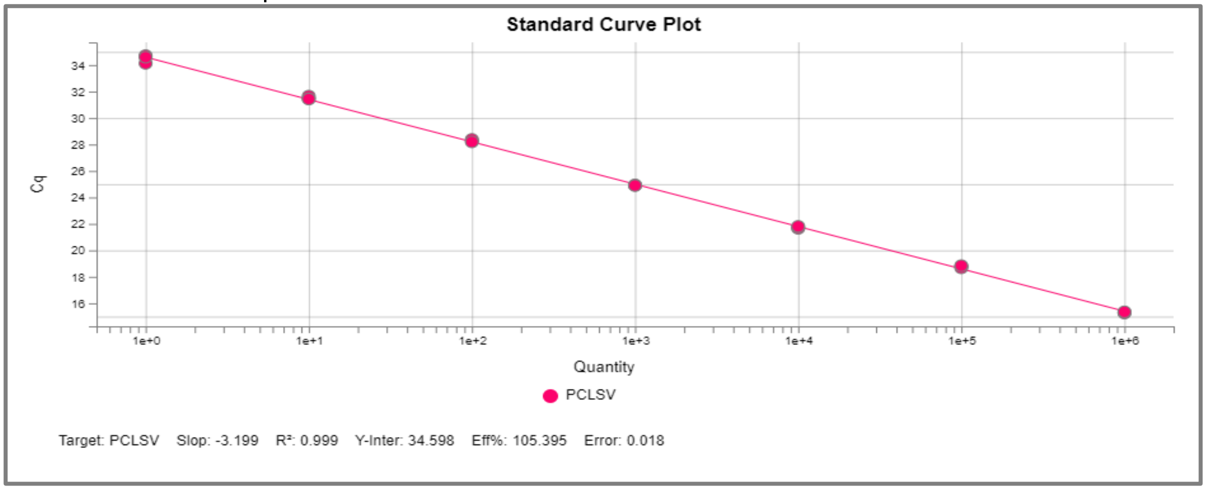

(M) Peach latent mosaic viroid

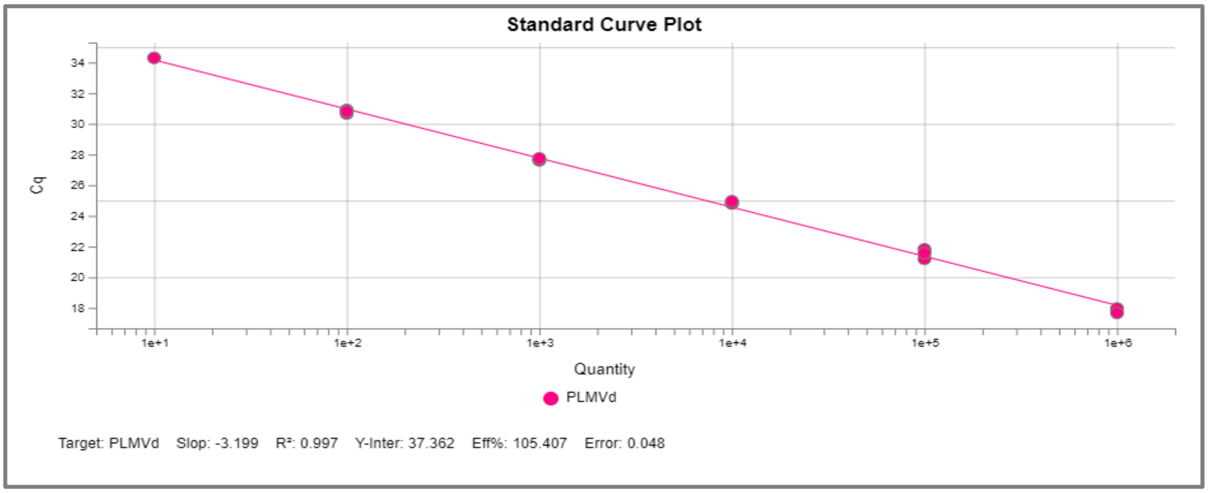

(N) Pear alphapartitivirus

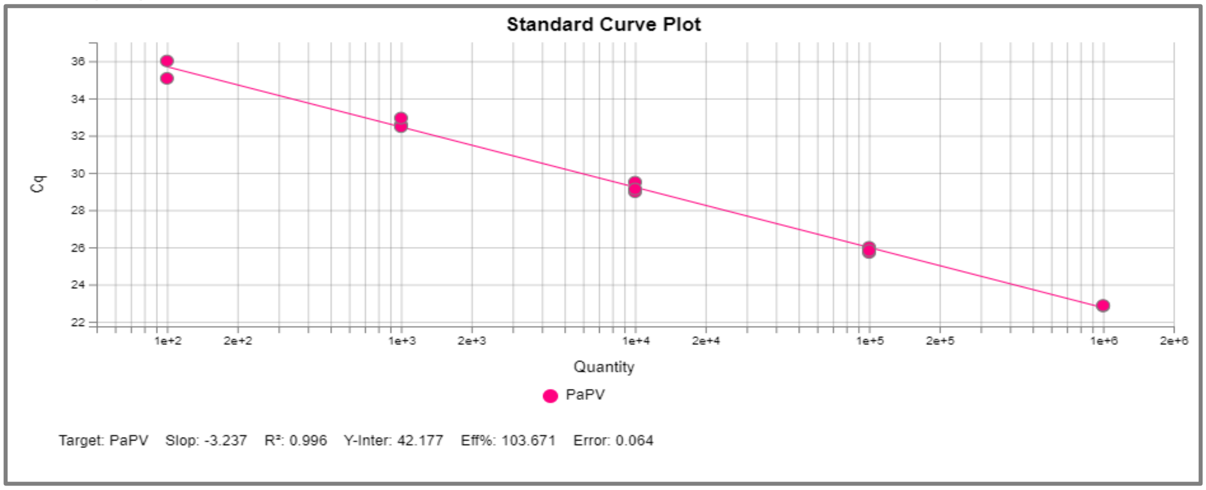

(O) Prunus Virus T

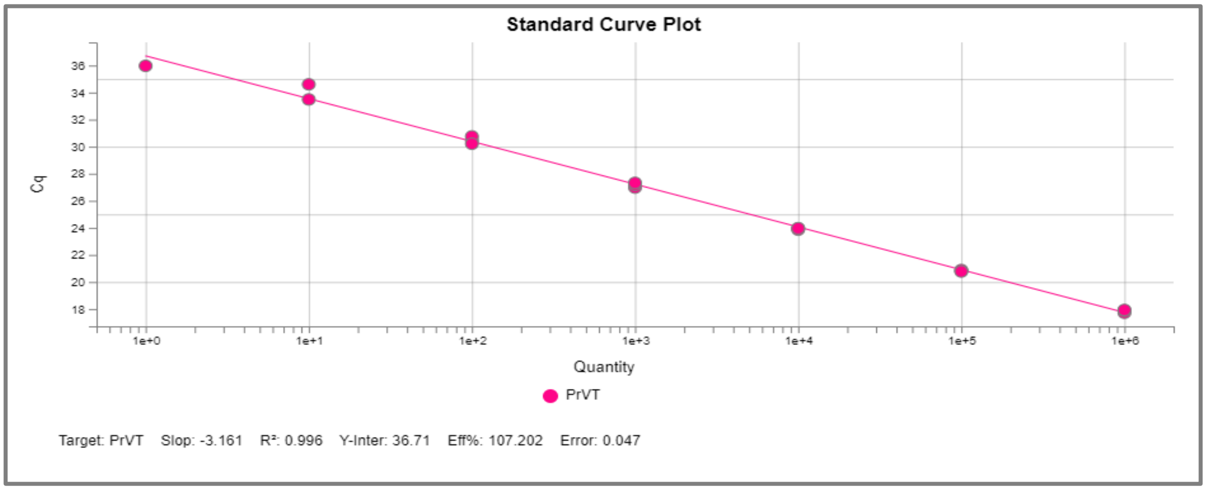

(P) *Phaseolus vulgaris* alphaendornavirus 1

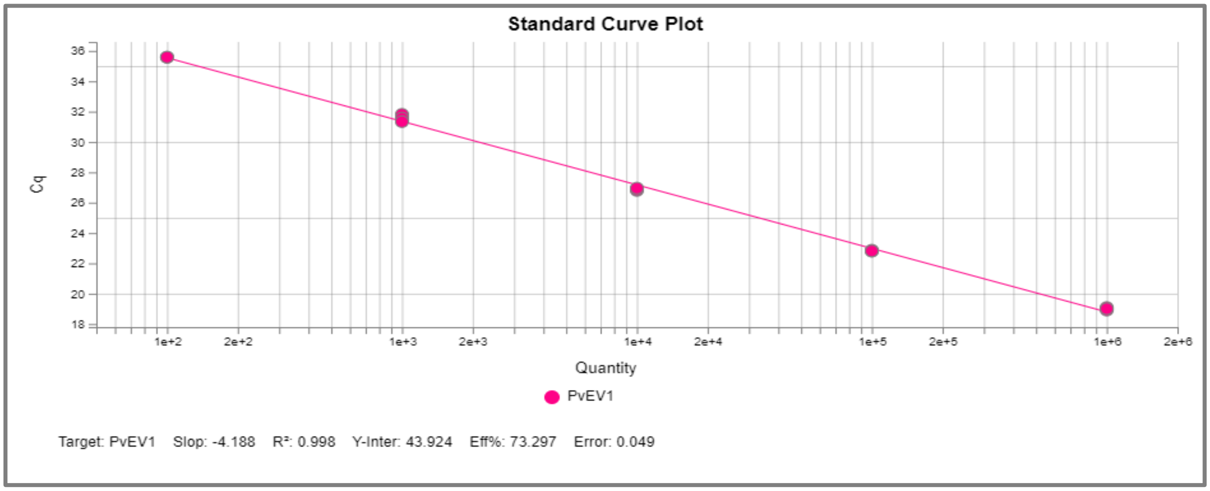

(Q) *Phaseolus vulgaris* alphaendornavirus 2

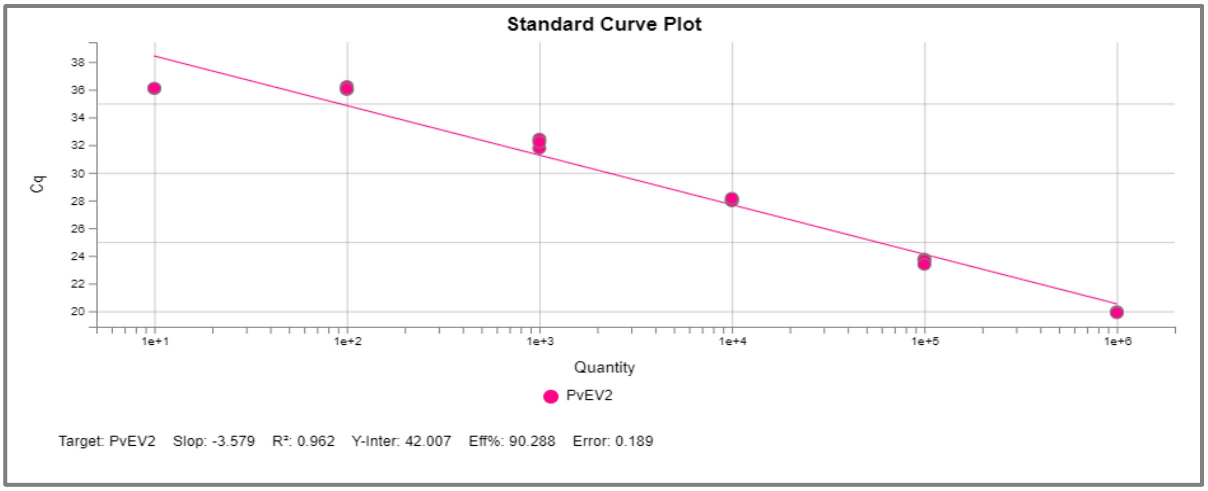

Supplement: Supplementary file 1 [file DataSheet_1.pdf]
